# Supplementary material for: Incorporation of the first and second heart fields and prospective fate of the straight heart tube via in vivo labeling of chicken embryos
Source: PLoS One. 2020 Jul 10;15(7):e0234069. doi: 10.1371/journal.pone.0234069 (PMC7351196; doi:10.1371/journal.pone.0234069)
Supplement: S1 Data — (DOCX) [file pone.0234069.s001.docx]

**Incorporation of the first and second heart fields and prospective fate of the straight heart tube via *in vivo* labeling of chicken embryos**

These author names are correct

Villavicencio Guzmán Laura^1^, Salazar García Marcela^1^, Jaime Cruz Ricardo^1,2^ , Lazzarini Roberto^3^, Toledano-Toledano Filiberto^4^, Sánchez Gómez Concepción^1^*

These are the correct assignments of the authors

1. Laboratorio de Investigación en Biología del Desarrollo y Teratogénesis Experimental, Hospital Infantil de México Federico Gómez, Mexico City, Mexico
2. Posgrado en Biología Experimental, Departamento de Ciencias de la Salud, Universidad Autónoma Metropolitana - Iztapalapa, Mexico City, Mexico
3. Departamento Biología de la Reproducción, División de Ciencias Biológicas y de la Salud, Universidad Autónoma Metropolitana-Iztapalapa, Mexico City, Mexico
4. Unidad de Investigación en Medicina Basada en Evidencias, Hospital Infantil de México Federico Gómez Instituto Nacional de Salud, Mexico City, Mexico

*Corresponding author

E-mail: [csgomez_2000@yahoo.com](mailto:csgomez_2000@yahoo.com); [csanchez@himfg.edu.mx](mailto:csanchez@himfg.edu.mx)

Short title: Developmental patterning of the first and second heart fields

**Abstract**

Recent discoveries of at least two heart fields and dynamic nature of cardiac development as well as controversies regarding the participation of heart fields in development of different heart structures led us to investigate the dynamics of incorporation of the first and second heart fields and prospective fate of the straight heart tube by labeling chicken embryos *in vivo* with the fluorescent lipophilic dye DiI. The cephalic and caudal limits of the anterior and posterior segments of the straight heart tube were labeled in two groups of embryos. Labels were tracked along the “C,” “S,” and “U” loops up to the tetracavitary or mature heart (n = 30 embryos/group; torsion and looping stage). To determine whether the atria and atrioventricular canal are derived from the first heart field the straight heart tube was cultured *in vitro* and immunodetection of Sox-9 and troponin I was performed to identify the mesenchymal and myocardial lineages respectively. Proliferating cell nuclear antigen (PCNA) immunodetection was used to determine the involvement of cell proliferation in heart tube development during torsion and looping. Embryological constitution of the straight heart tube and heart looping (C, S, and U) were not consistent with current descriptions. In fact, right ventricle precursors were absent in the straight heart tube derived from the first heart field. During torsion and looping, the cephalic segment of the straight heart tube gradually shifted into the heart tube until it was located at the myocardial interventricular septum in the tetracavitary heart. In contrast, the caudal segment of the straight heart tube was elongated and remodeled to become the first heart field derived left ventricle and the proximal part of the ventricular inlets. The ventricular outflows, right ventricle, distal part of the ventricular inlets, and atria developed from the second heart field.

**Introduction**

The heart is the first organ to function during embryonic development. It is mainly formed by contribution of two spatially and temporally overlapping cardiac progenitors arising from the splanchnic layer of the anterior lateral mesoderm forming the first heart field (FHF) and the second heart fields (SHF) [1]. FHF is initially distributed into two cardiogenic areas, situated at both sides of the midline of the embryo, whose cells migrate in the cephalomedial direction to form the cardiac crescent [2,3]. Folding of the cardiac crescent, toward the ventral midline, results in the formation of two primitive endocardial primordia covered by a myocardial mantle. Subsequently, both endocardial primordia coalesce along the ventral midline via “zipping” to form a myocardial primitive heart semi-tube [4,5], which is currently believed to arise from FHF [6]. During their migration as a cohesive sheet, cells of the heart region become epithelial and undergo cardiac differentiation, exhibiting organized myofibrils around the time of their fusion [3,7]. The remaining pharyngeal extended splanchnic mesoderm, which is initially located below the medial zone of the cardiac crescent is distributed below the cephalic, caudal, and lateral limits of the classically named straight heart tube, and corresponds to SHF, which continues to converge with the heart tube during torsion and looping [1,8-11]. The SHF splanchnic mesoderm, unlike that of FHF, is characterized by a high proliferation rate prior to its recruitment into the heart tube and delayed differentiation, which begins once the SHF cells are recruited into the heart tube [12]. The transcription factors Isl-1 and Tbx-1 as well as the fibroblastic growth factors Fgf8 and Fgf10 are the markers for SHF [9,13,14]. Recently, Kidokoro (2018) tracked the fate of splanchnic mesodermal cell populations in chicken embryos during heart tube formation and concluded that the early heart tube is formed by joint contribution of the lateral (primary) and middle (secondary) heart fields [11]. Moreover, they claimed that transformation of two-dimensional planar primordia into a three-dimensional structure occurs in close coordination with transformation of the adjacent endoderm into anterior intestine [11].

Classic descriptive studies in humans, mice, and birds have indicated that the primitive cardiac cavities were present in the straight heart tube, from which all anatomical components of the mature heart and great arteries are derived [15-17]. Subsequently, *in vivo* labeling with gelatin/India ink or the lipophilic DiI stain highlighted the contribution of several segments of the embryonic heart to conformation of the definitive cardiac chambers. De la Cruz et al. (1989) reported that the straight heart tube comprises the anterior segment (AS) and posterior segment (PS), which are delimited by the right and left interventricular grooves [4] or the right and left lateral furrows [18]. However, cardiac development analysis based on gradual recruitment of undifferentiated cells arising from the pharyngeal mesoderm or SHF to the arterial and venous poles of the linear heart tube revealed great discrepancies regarding embryonic components present in the straight heart tube and at each torsion and looping stage (“C”, “S”, and “U” loops). Moreover, the anatomical contribution of each embryonic cardiac segment to the mature heart remains controversial. Some studies found that most of the straight heart tube corresponds to the left ventricle (LV) primordium, with a small cephalic portion displaying right ventricle (RV) identity [19]. Alternatively, the straight heart tube has been assumed to comprise the primordial segments of LV and RV [4,20]. Another hypothesis postulates that LV is formed exclusively by contributions of the straight heart tube [5,19,21]. The anterior segment (AS) of the straight heart tube has been proposed to be composed of the LV primordium, with the two caudal arms representing the left and right atrial chambers precursors [12,22,23]. Furthermore, there is no consensus regarding contributions of the SHF pharyngeal mesoderm at the cephalic and caudal limits of the heart tube. Dil labeling in mouse and chick embryos and molecular analyses of cell lineages in transgenic mice demonstrated that cell populations entering the cephalic limit of the heart tube are involved in the development of RV and outflow tracts [13,19,24-27]. However, the stages at which RV precursor recruitment is completed and embryonic outflow (conus and truncus) incorporation is initiated remain unknown. Through analysis of Isl1 gene expression patterns in the dorsal pharyngeal mesoderm, Cai (2003) demonstrated the extensive contribution of SHF to the mouse developing heart, including ventricular outflows, RV, a small part of LV, and large proportions of the atria [13]. Involvement of the caudal SHF in development of the atrioventricular canal and primitive atria [6,23,27-29] or the primitive atria and sinus venous [27] has also been noted. However, using the mef2c-AHF-Cre transgenic mice, Verzi et al. (2005) did not find any evidence of contribution of the anterior SHF to the development of the atria or LV [25]. More recently, by labeling and tracking Mesp1+ cells in the mouse gastrula, Devine et al. (2014) reported that during gastrulation, cardiac precursors are specified in two transcriptionally different cardiac populations separated by the interventricular septum precursors [30]. One group (presumptive FHF) forms the atria and LV, whereas the other (putative SHF) forms RV and “outflow tract” which may further form the great vessels [30]. In fact, at present, there is no generalized agreement regarding whether the primitive atria are formed from FHF or SHF. Finally, the cellular source of the myocardial interventricular septum precursors has received very little attention and remains an unresolved issue. Pioneer *in vivo* labeling studies with gelatin/India ink in birds have identified an area confined to the ventral fusion line of both cardiac primordia at the level of interventricular grooves as the precursor of middle and apical thirds of IVS [4,31,32]. More recently, genetic and molecular studies have identified joint contribution of FHF and SHF in IVS embryogenesis [33]. In addition, via clonal genetic fate mapping in mice, Devine et al. (2014) identified the supposed IVS precursor between two ventricular precursors at the beginning of gastrulation [30].

Based on the discovery of FHF and SHF, dynamic nature of cardiac development, and previously mentioned controversies regarding the embryological composition of the straight heart tube, this study aimed to determine the dynamics of the incorporation of FHF and SHF and prospective fate of the FHF-derived straight heart tube in chicken embryos using *in vivo* labeling. Topological changes in the heart tube during torsion and looping were examined, and *in vitro* explant assays were performed. Sox-9, troponin I, and proliferating cell nuclear antigen (PCNA) immunostaining was used to identify the mesenchymal or myocardial lineage, and cell proliferation, respectively. Contrary to the current data, we reported that AS of the straight heart tube contributes to IVS development, while PS is involved in the development of LV and ventricular inlets but not the atria. The information obtained in this research provides a frame of reference for molecular approaches to the origin of primitive cardiac segments and lays a foundation for future studies focused on the origin of different congenital heart diseases involving the cardiac ventricles and IVS.

**Materials and Methods**

**Eggs**

A total of 900 Bovans chicken eggs were obtained from the ALPES local poultry farm (Tehuacan, Pue, Mexico). The eggs were incubated at 37°C in a humid atmosphere and automatically turned until reaching the desired stage. The embryonic age was established according to the Hamburger and Hamilton schedule [34]. Of the 900 fertile eggs purchased, only 330 eggs were used to acquire embryos displaying a normal morphology and hearts with no apparent defects, which were distributed as follows: Two hundred and forty embryos were used to examine the prospective fate of the straight heart tube by *in vivo* labeling. Moreover, 60 embryos were used to PCNA immunostaining (n = 30) and analyze topological changes in the heart tube during torsion and looping (n = 30). Thirty additional eggs were used to obtain HH10 embryos for *in vitro* explant assays and Sox-9 and troponin I immunostaining. The remaining fertile eggs were discarded because they were unviable, did not reach the correct age for labeling, died during manipulation, or showed abnormal development. The animal use protocols and all experimental procedures were strictly based on the Mexican Official Guideline (NOM-062-ZOO-1999). In addition, the research, ethics, and biosafety committees of Children’s Hospital of Mexico Federico Gomez approved this project (HIM-2016-051).

***In vivo* labeling**

To study the development of AS and PS of the straight heart tube through torsion and looping (HH10–16) and determine their contribution to the tetracavitary heart (HH34) via *in vivo* labeling, chicken embryos were divided into eight groups, each with 30 normal embryos (Table 1). In this study, we used mature heart (HH34) to name the tetracavitary organ in which the cardiac embryonic structures have been transformed into definitive anatomical structures. For technical reasons, the labeling experiments were performed using two types of culture. The first period, covering torsion and looping (HH10 to HH16), involved *in vitro* culture [35] using six groups: G1a, G1b, G1c, G3a, G3b, and G3c (Table 1). The second period, spanning development from the U loop to the mature heart (HH16-34) involved *in ovo* culture as described by de la Cruz et al. (1977, 1989) [4,36] and using two groups (G2 and G4) (Table 1),

**Table 1. Study Group Features and Embryos Acquired.**

| **Group** | **Cardiac segment** | **Dye labeling** | | **Culture technique** | **Normal embryos acquired for use in several studies (n)** |
| --- | --- | --- | --- | --- | --- |
|  |  | **Initial stage** | **Terminal development** |  |  |
| **G1a** | Anterior (cephalic and caudal limits) | Straight heart tube (HH10) | C loop (HH12) | *In vitro* | 30 |
| **G1b** | Anterior (cephalic and caudal limits) | C loop (HH12) | S loop (HH14) | *In vitro* | 30 |
| **G1c** | Anterior (cephalic and caudal limits) | S loop (HH14) | U loop (HH16) | *In vitro* | 30 |
| **G2** | Anterior (cephalic and caudal limits) | U loop (HH16) | Mature heart (HH34) | *In ovo* | 30 |
| **G3a** | Posterior (cephalic and caudal limits) | Straight heart tube (HH10) | C loop (HH12) | *in vitro* | 30 |
| **G3b** | Posterior (cephalic and caudal limits) | C loop (HH12) | S loop (HH14) | *in vitro* | 30 |
| **G3c** | Posterior (cephalic and caudal limits) | S loop (HH14) | U loop (HH16) | *In vitro* | 30 |
| **G4** | Posterior (cephalic and caudal limits) | U loop (HH16) | Mature heart (HH34) | *In ovo* | 30 |
|  |  |  |  | **Total** | 240 |

**Preparation of filaments for labeling**

Fine glass filaments (10 μm × 10 cm) were varnished with a liquid gelatin/water (5%) mixture. After solidification of the gelatin, the prepared filaments were stored in a sterile Petri dish in a refrigerator [37].

**Labeling via *in vitro* culture**

To study development of the AS of the straight heart tube during torsion and looping (HH10–16), the hearts of embryos were exposed by creating longitudinal cuts at the vitelline and pericardial membranes. To delimit AS of the straight heart tube, a small piece (0.5 mm) of the previously prepared glass filament embedded in the lipophilic fluorescent dye DiI (Molecular Probes, V22889) was gripped at the pharyngeal mesoderm adjacent to the cephalic limit of the straight heart tube. Another filament was gripped at the fusion line of the two heart primordia at the level of the putative interventricular grooves. To study development of PS of the straight heart tube, the labels were gripped onto the pharyngeal mesoderm, located at the distal left and right borders of the heart. In both cases, the glass filaments were removed after 30 seconds. The embryos were incubated at 37.5°C in a moist atmosphere for a sufficient duration (between 24 and 36 hours) to track the labels through St HH10–12 (C loop; G1a and G3a), St HH12–14 (S loop; G1b and G3b), and St HH14–16 (U loop; G1c and G3c). The final position of the fluorescent labels was determined using a stereomicroscope and an Axiocam MRC digital camera under clear and dark fields with a rhodamine filter. Images were obtained at 80× magnification.

**Labeling by *in ovo* culture**

To determine the anatomical contribution of AS (**Group 2**) and PS (**Group 4**) of the straight heart tube to development from the U loop (HH16) through the tetracavitary heart (HH34), the embryos were labeled and cultured *in ovo*. In order to place the label, a window, measuring approximately 1 cm^2^, was opened in the egg shell. After exposing the heart (HH16) by dissecting the vitelline and pericardial membranes, a small piece (0.5 mm) of the previously prepared glass filament embedded in Dil was adhered to the myocardium in the areas at which the tracked labels were located in the *in vitro* analysis. In both groups (G2 and G4), the shell window was covered with an adhesive tape, and the eggs were incubated at 37°C undersaturated humidity until development of the tetracavitary mature heart (HH34). The final position of the fluorescent labels was determined as described in the *in vitro* culture. In contrast, to identify the position of the fluorescent labels in the internal structures of the heart, eight fixed mature hearts were dehydrated and embedded in PEG1400, as described by Lazik et al. (1997) [38]. Histological sections (8μm) of the heart were obtained and observed under an Axiovert 100M confocal microscope (Carl Zeiss).

**Scanning electron microscopy**

Scanning electron microscopy was used to determine anatomical and topographic changes in the cephalic and caudal segments of the straight heart tube during torsion and looping. A total of 30 eggs were used to acquire embryos at stages HH10, HH12, HH14, HH16, HH24, and HH34. The embryos were perfused with 2.5% glutaraldehyde in phosphate-buffered saline (PBS; PH 7.4) and maintained for 2 hours in the same fixative at room temperature. Upon dissecting from the embryos, the hearts were osmicated (1% OsO_4_ in PBS) for 1 hour, dehydrated in a graded ethanol series (30%–100%), and dried in a critical point apparatus (Samdri 789A, Tousimins Research Co.). Finally, the specimens were coated with a thin gold monolayer using an iron sputtering apparatus (Denton Vacuum Desk 1A). Photographs were captured using a JSM 5300 scanning electron microscope (Jeol).

***In vitro* explants assays**

To investigate whether the caudal limit of PS of the straight heart tube includes mesenchymal (AV canal precursors) or myocardial (atrial) lineages, thirty FHF-derived straight heart tubes attached to the ventral wall of the anterior portal intestine were explanted and cultured *in vitro*. The samples were cultured in a ringer/fluid albumin solution (2:1) for 15 hours. At the beginning and end of the *in vitro* explants assays, photographs were obtained using a Carl Zeiss stereomicroscope and an Axiocam MRC camera. Finally, 10 cultured explants were processed for Sox-9 and troponin I immunodetection.

**Heart growth**

PCNA immunostaining was performed to investigate if the FHF-derived straight heart tube grows via hypertrophy rather than proliferation and the SHF-derived cell population proliferation when incorporated into the cardiac tube. The immunostaining was performed using straight heart tube and C, S, and U loops.

**Immunofluorescence**

Deparaffinized histological sections of heart explants and hearts intended for cell proliferation analysis were rehydrated in a graded ethanol series (100% – 30%) and immersed in distilled water, followed by PBS (pH 7.4) and an antigen retrieval solution (DAKO Target Retrieval Solution S2369). After being exposed to pressure at 103.4kPa (15 psi) for 5 minutes, tissue sections were washed in PBS, followed by immersion in the Protein Block Serum-Free solution (Dako X0909). To identify mesenchymal tissue (putative canal AV or inlet precursor) and myocardium (putative atrium) in the new vesicle formed at the caudal limit of the straight heart tube explants, histological sections were incubated with a combination of troponin I (goat polyclonal IgG, SC 8118) and FITC-conjugated anti-goat secondary antibody (SC 2024) with green fluorescence and a combination of Sox-9 (mouse polyclonal IgG, SC 166505) and rhodamine-conjugated secondary antibody (Sc 2368) with red fluorescence. Cell nuclei were counterstained with Red Dot II (Biotium 40061-1) with blue fluorescence. Images were acquired using a laser-scanning confocal microscope system (Axiovert 100M). For PCNA immunostaining, histological sections of embryonic hearts at different stages of torsion and looping (straight heart tube, C, S, and U loops) were incubated with anti-PCNA antibody (sc-56) and rhodamine-conjugated goat anti-mouse IgG-R (sc-2092) with red fluorescence. Cell nuclei were contrasted with 4′,6-diamidino-2-phenylindole (Sigma Aldrich D9542) with cyan fluorescence. Images were acquired with a laser-scanning confocal microscope system (LSM-780 NLO microscope). For both experiments, only secondary antibodies were used as controls for immunofluorescence.

**Numerical data analysis**

The final location of Dil fluorescent labels at each period of the study and the size of the cardiac vesicle formed at the caudal end of the explanted hearts were recorded, and the percentage was calculated. Results were graphically represented using Microsoft Excel. For proliferation analysis, cells with PCNA-positive nuclei were quantified in six panoramic images of the histological sections at each stage, and the percentage of labeled cells with respect to the total number of cells comprising each heart fields and the standard deviation were calculated.

**Results**

To determine the fate of the straight heart tube and developmental dynamics of AS and PS of straight heart tube, a small group of DiI-labeled cells were examined during torsion and looping via *in vitro* culture and then traced to the mature heart via *in ovo* culture (Figs 1 and 2; Tables 2 and 3).

**Involvement of the Anterior Segment of the Straight Heart Tube in the Development of Apical and Medial Thirds of the Interventricular Septum**

Representative images tracing the cephalic and caudal limits of AS of the straight heart tube were acquired using bright field and fluorescence microscopy. The incidence of labeling on structures of the embryonic heart is presented in Fig 1 and Table 2. AS of the straight heart tube gradually displaced toward the embryonic ventricular region during torsion and looping of the heart tube. In the C-looped heart the tube (HH12), formed the ascending or cephalic branch of the ventricular loop (putative primordium of the RV) and was located between the conoventricular grooves and the junction of the embryonic LV and RV or the great curvature (A, A’ compared with B, B’ in Fig 1). Most labels at the cephalic limit of AS of the straight heart tube (93%) were displaced along the distal region of the ascending branch of the heart loop, whereas almost all labels at the caudal limit of AS of the straight heart tube (97%) appeared in the zone of the great curvature of the loop (Table 2), considered by de la Cruz et al. (1997, 1998) to be the putative primitive IVS precursor [31,32]. Subsequently, in the S-looped heart (HH14), AS of the straight heart tube maintained its location in the ascending branch of the heart loop (A, A’ compared with C, C’ in Fig 1). It extended from the zone adjacent to the so-called conoventricular grooves, where the cardiac tube begins to flex into a ventrodorsal direction (93% labels from the cephalic limit) and the middle point of the great curvature of the heart (87% labels from the caudal limit) (Table 2). Once the cardiac tube reached the U loop (HH16), both branches of the cardiac loop occupied a lower position side by side and displayed incipient trabeculations. The originally named ascending branch (putative RV primordium) occupied the right position (Fig 1C, C’) between what are now known as the conoventricular grooves (74% labels from the cephalic limit), and which were named by de la Cruz et al. [31,32] the IVS primordium (93% labels from the caudal limit). In contrast, the descending branch or putative LV primordium occupied the left position. Interestingly, the cardiac precursors initially located between the cephalic and caudal limits of AS of the straight heart tube, which were displaced during torsion and looping to be the putative RV primordium basically converged on the ventricular apex in tetracavitary hearts (Fig 1D, D’). However, labels at each limit were distributed differently (Table 2). Labels of the cephalic limit were scattered along the myocardial wall of the trabeculated region of RV. Moreover, 47% labels appeared as small fluorescent groups dispersed near the limit with the outflow. In addition, 53% labels at the cephalic limit and all labels at the caudal limit formed a near compact fluorescent group at the apex of RV (Fig 1E,E’ and Table 2). Notably, histological sections of the mature hearts (n= 8) exhibited extremely scattered labels along the myocardium of IVS from its middle third to the right surface of the apex (Figs 1F, F′). In two cases, labels at the caudal limit of AS of the straight heart tube were the closest to the basal third of IVS, whereas those at the cephalic limit of AS of the straight heart tube only reached the middle third of IVS.

**Fig 1. Anterior Segment of the Straight Heart Tube is Involved in the Development of the Apical and Medial Thirds of the** **Interventricular Septum.** (A, A’) Bright field and fluorescent microscopy images of the straight heart tube with the anterior segment delimited by the cephalic (blue arrow) and caudal (green arrow) labels. (B, B’–D,D’) C-, S-, and U-looped hearts. Note the anterior segment of the straight heart tube displaced to the supposed right ventricular primordium. The cephalic label (blue arrow) is located in the distal region of the ascending branch of the loop near the limit between the conus and the right ventricle primordium. The caudal label (green arrow) is located at the midpoint of the great curvature, at the level of the supposed interventricular septum precursor. (E, E’) Dorsal view of a tetracavitary heart (HH34) revealing the labels at both limits of the straight heart tube converging at the right ventricular apex. (F, F’) Histological sections of a tetracavitary heart showing the caudal label (green arrow) immersed in the myocardium at the middle third of IVS. Abbreviations: AV, atrioventricular; C, conus; LA, left atrium; LV, left ventricle; LVp, left ventricle primordium; PRA, primitive right atrium; PLA, primitive left atrium; RA, right atrium; RV, right ventricle.

**Table 2. Displacement of Labels from the Cephalic and Caudal Limits of the Anterior Segment of the Straight Heart Tube to the Interventricular Septum.**

**TRACKING OF THE ANTERIOR SEGMENT OF THE STRAIGT HEART TUBE**

| **TRACKING PERIOD** | **HH10-12** | | **HH12-14** | | **HH14-16** | | **HH16-34** | |
| --- | --- | --- | --- | --- | --- | --- | --- | --- |
| **EMBRYONIC SEGMENT/** | CEPHALIC LIMIT | CAUDAL LIMIT | CEPHALIC LIMIT | CAUDAL LIMIT | CEPHALIC LIMIT | CAUDAL LIMIT | CEPHALIC LIMIT | CAUDAL LIMIT |
| **CVSul** | 2 (7%) | 0% | 0% | 0% | 0% | 0% | _ | _ |
| **DRABL (RVp)** | 28 (93%) | 0% | 28 (93%) | 0% | 7 (23%) | 0% | _ | _ |
| **PRABL** | 0% | 0% | 2 (7%) | 0% | 22 (74%) | 0% | _ | _ |
| **IVSul/pIVS** | 0% | 29 (97%) | 0% | 26 (87%) | 1 (3%) | 26 (93%) | _ | _ |
| **LVp** | 0% | 1 (3%) | 0% | 4 (13%) | 0% | 2 (7%) | _ | _ |
| **ANATOMIC STRUCTURE** |  | | | | | | | |
| **RVOFT/ TRRV** | _ | _ | _ | _ | _ | _ | 14 (47%) | 0% |
| **Ventricular apex** | _ | _ | _ | _ | _ | _ | 4 (13%) | 0% |
| **IVS** | _ | _ | _ | _ | _ | _ | 12 (40%) | 30 (100%) |
| **TOTAL (n)** | 30(100%) | | 30(100%) | | 30(100%) | | 30(100%) | |

**Embryonic segments**: CVSul, conoventricular sulcus; DRABL, distal region of the ascending branch of the loop; PRABL, proximal region of the ascending branch of the loop; IVSul, interventricular sulcus; PIVS, primitive interventricular septum; LVp, left ventricle primordium; RVp, right ventricle primordium. **Anatomical Structures**: RVOFT, right ventricle outflow; TRRV, trabecular region of the right ventricle; IVS, interventricular septum. Blue and green shading are representing the color of the cephalic and caudal labels in figure 1.

**Involvement of the Posterior Segment of the Straight Heart Tube in the Development of the Atrioventricular Canal and Left Ventricle**

Representative bright field and fluorescence microscopy images of tracking of the prospective fate of PS of the straight heart tube and incidence of labeling of each embryonic heart structures are shown in Fig 2 and Table3. Cell tracking of the distal left and right ends of PS of the straight heart tube revealed that this originally bifurcated cardiac segment acquired the shape of a single tube in the C loop. PS of the straight heart tube extended along the caudal branch of the cardiac loop from the middle zone of the great curvature or interventricular groove (cephalic limit) to the adjacent region of so-called left and right AV grooves (caudal limit). Most labels at the left (80%) and right (67%) distal ends of PS of the straight heart tube were located at the AV grooves. In some cases (20% left and 33% right ends), the labels reached the bifurcated region considered an atrial lineage (Table 3; A, A’ compared with B, B’ in Fig 2). In the S loop, PS of the straight heart tube was incorporated along with the currently known embryonic LV. In most cases (left 77% and right 80%), cell population at the caudal limits of PS of the heart tube remained in the vicinity of AV canal (Fig 2C and Table 3). The rest of these labels (23% and 20%, respectively), were located in the supposed atria. In the U loop, PS of the straight heart tube remained on the dorsal and ventral sides of the presumed embryonic LV, which was more clearly observed from a left view (Fig 2D, D’). The cardiac precursors originally located at the cephalic limit of PS of the straight heart tube (HH10) shared the same fate as those located at the caudal limit of AS of the straight heart tube (Tables 2 and 3). Cell populations at the left (93%) and right (93%) borders of PS of the straight heart tube reached the AV canal, and the remaining labels (7% and 7%, respectively) appeared in the putative atrial region (Table 3). However, in the mature heart (HH34), these cell populations were incorporated into the ventral (97%) and dorsal (83%) walls of the AV canal (D compared with D’,E, E’,F, and F´ in Fig 2 and Table 3). In some cases the labels were located surrounding walls of the AV annulus and atria (3% and 17%, respectively, Table 3).

**Fig 2. Posterior Segment of the Straight Heart Tube is Involved in the Development of the Atrioventricular Canal and Left Ventricle.** (A, A’) Straight heart tube with a label on the distal end of the posterior segments of the right (yellow arrow) and left (red arrow) branches. (B,B’) C-looped heart. Note the tubular shape of the proximal region of the posterior segment of the straight heart tube. The right and left caudal regions, which are supposed to be the primitive atria, remain bifurcated. (C, C’ and D, D’) S- and U-looped hearts. Note that the originally bifurcated posterior segment of the straight heart tube acquired a tubular shape. Labels at the right and left borders of the posterior segment of the straight heart tube converged at the currently named atrioventricular grooves, adjacent to the atrioventricular canal. (E, E’ and F, F’) Ventral and dorsal views of a mature heart. Observe labels at the level of the ventral (red arrow) and dorsal (yellow arrow) atrioventricular canal wall. Abbreviations: AV, atrioventricular; C, conus; LA, left atrium; LV, left ventricle; LVp, left ventricle primordium; PRA, primitive right atrium; PLA, primitive left atrium; RA, right atrium; RV, right ventricle. Dotted line represents the interventricular septum.

**Table 3. Displacement of Labels from the Cephalic and Caudal Limits of the Posterior Segment of the Straight Heart Tube to the LV and AV Canal.**

**TRACKING OF THE POSTERIOR SEGMENT OF THE STRAIGT HEART TUBE**

| **TRACKING PERIOD** | **HH10-12** | | | **HH12-14** | | | **HH14-16** | | | | **HH16-34** | | | |
| --- | --- | --- | --- | --- | --- | --- | --- | --- | --- | --- | --- | --- | --- | --- |
| **EMBRIONIC SEGMENT** | CEPHA LIMIT | CAUDAL LEFT | CAUDAL RIGT | CEPHA LIMIT | CAUDAL LEFT | CAUDAL RIGT | | CEPHA LIMIT | CAUDAL LEFT | CAUDAL RIGT | | CEPHA LIMIT | CAUDAL LEFT | CAUDAL RIGT |
| IVSul/IVSp | 29(97%) | 0% | 0% | 26 (87%) | 0% | 0% | | 28(92%) | 0% | 0% | | 0% | 0% | 0% |
| LVp | 1 (3%) | 0% | 0% | 4 (13%) | 0% | 0% | | 2 (7%) | 0% | 0% | | 0% | 0% | 0% |
| AVCanal | 0% | 24 (80%) | 20 (67%) | 0% | 23 (77%) | 24 (80%) | | 0% | 28 (93%) | 28 (93%) | | 0% | 0% | 0% |
| Putative Prinitive Atria | 0% | 6 (20%) | 10 (33%) | 0% | 7 (23%) | 6 (20%) | | 0% | 2 (7%) | 2 (7%) | | 0% | 0% | 0% |
| ANATOMIC STRUCTURE |  |  |  |  |  |  | |  |  |  | |  |  |  |
| IVSul | _ | _ | _ | _ | _ | _ | | _ | _ | _ | | 30 (100%) | 0% | 0% |
| LV to AVCanal (inflow) | _ | _ | _ | _ | _ | _ | | _ | _ | _ | | 0% | 29 (97%) | 25 (83%) |
| AV anuulus/ Atria | _ | _ | _ | _ | _ | _ | | _ | _ | _ | | 0% | 1 (3%) | 5 (17%) |
| TOTAL (n) |  | 30 (100%) | |  | 30 (100%) | | |  | 30 (100%) | | |  | 30 (100%) | |

**Embryonic segments:** AV annulus, atrioventricular annulus; pIVS, primitive interventricular septum; LVp, left ventricle primordium. **Anatomical Structures:** AVC, atrioventricular canal; IVS, interventricular septum; IVSul, interventricular sulcus; LV, left ventricle. Green, red and yellow shading are representing the color of the cephalic and caudal labels in figures 1 and 2.

**Atria did not Arise from the First Heart Field-derived Straight Heart Tube**

After 15 hours of *in vitro* culture, the explanted straight heart semi-tube (HH10) formed a C-looped heart tube in all cases despite the lack of SHF (n = 20). In addition, a small (80%) or slightly large (20%) vesicle developed at the caudal limit of the heart tube (Figs 3A, D). Immunostaining showed the presence of cytoplasmic troponin I (green fluorescence) and Sox-9 (red fluorescence) in cells along the C-looped heart (Fig. 3C). In contrast, troponin I was absent in most cells bordering the newly formed vesicles, while Sox-9 was abundantly expressed in the nuclei of these cell (Fig. 3C’).

**Fig 3. Representative Images of the *In Vitro* Explants Assays Showing that the Atria did not Arise from the First Heart Field-derived Straight Heart Tube.** (A and B) Recently explanted straight heart semi-tubes (HH10). (A’ and B’) The same hearts after 15 h of *in vitro* culture. The C-looped heart tubes and a new vesicle formed at their caudal limit. (C) Panoramic view of a C loop showing the presence of cytoplasmic Troponin I (green fluorescence) and Sox-9 (red fluorescence) in cells along the cardiac tube. (C’) Newly formed cardiac vesicles at the caudal end of C loop comprising cells expressing nuclear Sox-9 alone.

**PCNA detection indicates that cell populations derived from FHF show less proliferation than those derived from SHF**

Figure 4 reveals results of the analysis of the distribution patterns of proliferating cells using PCNA expression in serial sections of embryonic hearts during torsion and looping. In the straight heart tube (FHF) only 20% of the cells were positive for PCNA (Fig. 4A, A’). Among HH12–14, cell populations of both cardiac fields showed a double increase in the proliferation recording 40% of PCNA positive cells (Fig. 4B, B’, C,C’, F). In U-loop (Fig. 4D, D’; E,E’); a peak of 50% and 60% of PCNA expression is observed in cell populations derived from FHF and SHF (Fig. 4F).

**Fig 4. PCNA Distribution Patterns in the Cardiac Tube throughout Torsion and Looping.** (A, A′) Frontal view of a section of a straight heart tube (HH10). A small number of PCNA-positive fluorescent red cell nuclei are observed in the FHF delimited by two yellow dotted lines; (B, B’) Frontal view of a section of a C looped heart (HH12); (C, C’) left sagittal view of an S looped heart (HH14). Increased number of PCNA-positive fluorescent red cell nuclei are observed in cardiac segments derived from SHF; (D, D’). Right and left views of a section of a U looped heart (HH16). Notable increase in PCNA-positive fluorescent red cell nuclei in heart structures derived from both the first and second heart fields. (E) Graphical representation of the percentage of PCNA-labeled cells with respect to the total number of cells constituting each heart field at torsion and looping stages. Abbreviations: FHF, first heart field; SHF, second heart field.

**Discussion**

Morphological analysis, *in vivo* labeling, and molecular studies conducted over the last 20 years have generated a highly dynamic vision of cardiac development. The straight heart tube develops from FHF, whereas ISLT1-positive cell populations, which arise from SHF, are gradually incorporated at the cephalic and caudal limits of the straight heart tube to form the rest of the cardiac primordia. However, contributions of FHF-derived straight heart tube and cell populations arising from SHF to mature heart development remain currently controversial; therefore, this study tracked the fate of FHF-derived straight heart tube via *in vivo* labeling. Dynamic incorporation of the pharyngeal mesoderm (SHF precursor) at each torsion and looping stage(C-, S-, and U-looped hearts) was also examined. Contrary to the current knowledge, we found that AS of the straight heart tube contributes to IVS development. PS is involved in the development of the complete LV and a part of the ventricular inlets; however, it does not participate in atrial development. Based on the developmental dynamics of FHF and SHF, we are proposing a new developmental pattern of the heart tube during torsion and looping process, different from the universally accepted concept at present.

**AS of the straight heart tube is precursor of the myocardial IVS**

In agreement with de la Cruz et al. [4], tracking of AS of the straight heart tube during torsion and looping (HH12–16) allowed us to observe its gradual shift to the supposed RV primordium. This cardiac segment, delimited by two fluorescent labels, was located from the distal region of the ascending branch of the loop (adjacent to the conoventricular groove) to the middle region of the great curvature of the loop. Once the cardiac tube reached the U loop (HH16), the labels originally placed at caudal limit of AS of the straight heart tube were located between both branches of the loop (Fig. 1D,D’), which were considered by de la Cruz et al. to be IVS primordia [31,32]. However, in tetracavitary hearts (HH34), the labels at both limits of AS of the straight heart tube converged at the RV apex (Fig. 1E,E’). Although these results highlight the gradual incorporation of SHF-derived cell populations into the heart tube, these actually deny that AS of the straight heart tube corresponds to the RV primordium in chick embryos. Therefore, we assert that the RV primordium in birds, similar to what happens in mammals, arises from SHF [1,19,21]. In addition, some histological sections exhibited highly scattered fluorescent labels along the middle and apical thirds of IVS, which led us to conclude that the entire AS of the straight heart tube is indeed precursor of the myocardial IVS and not just the zone of the ventral fusion line of the cardiac primordia [31]. Pioneer studies in humans [39, 40], *in vivo* labeling in birds [41-44] and retrospective clonal analysis in mouse [45] support the importance of myocardial walls of the embryonic ventricles in IVS development.

**PS of the straight heart tube contributes to the development of the LV and AV canal**

Labeling of the cephalic and caudal limits of PS of the straight heart tube revealed that from formation of the C loop, the originally bifurcated cardiac segment acquires the shape of a single tube (Fig. 2B, B’). During torsion and looping (C–U loop), the cephalic limit of PS of the straight heart tube was located at the middle zone of the great curvature of the loop or the interventricular groove, whereas the caudal limit was adjacent to the region of the atrioventricular grooves (Fig. 2B–D). In the mature heart (HH34), the caudal limit of PS of the straight heart tube was located at the left atrioventricular annulus (Fig. 2E, E’), whereas the cephalic limit of PS of the straight heart tube located in the interventricular sulcus, similar to caudal limit of AS of the straight heart tube. Some mark residues initially placed on the caudal edge of the PS in the putative atrial region were found near AV annulus in three cases; therefore, it was necessary to define with certainty the possible involvement of PS in the development of the AV canal or formation of the primitive atria. Thus, we explanted straight heart tubes and cultured them in vitro. After 15 hours of culture, a C-looped heart tube was formed with a vesicle at the caudal border (Figs. 3A, D). Furthermore, immunofluorescence revealed that the C-looped heart was formed by myocardial cells expressing troponin I, as expected of the precursors of IVS and LV. In contrast, the newly formed vesicle was bordered by mesenchymal lineage with cells expressing nuclear Sox-9 (Fig. 3E, F). These results lead us to conclude more accurately that PS of the straight heart tube shows a dual fate; while its proximal region forms LV; its distal region participates in AV canal development. Our labeling experiments of both heart fields did not rule out the collective participation of LV and RV trabeculations, as observed by Franco, et al. (2006) in mice [43] and Contreras-Ramos (2008) in chickens [44]. However, our findings are contrary to certain developmental models of cardiac tube using transgenic mice, which suggested that the straight heart tube is destined to become the LV [1,13,19], and those indicating that the straight tube heart gives rise to the LV, atrioventricular canal, and atria [20].

**Detection of PCNA indicates that proliferation and hypertrophy may be involved in the development of the early cardiac tube.** Elongation of the early heart tube during torsion and looping has been attributed to gradual recruitment of the SHF splanchnic mesoderm. This vision seems to deny the importance of proliferation in the development of the FHF-derived straight heart tube. In contrast, proliferation has been considered important for formation of SHF-derived structures [11]. Our results of PCNA immunostaining in early heart tube (HH10–12) revealed weak proliferative rate (22%–23%); in contrast at later stages (HH14-16), when the AV canal is forming we found that cell proliferation rate doubled (Fig. 4). These data suggest that that the FHF derived straight heart tube is initially formed by hypertrophy and some proliferation, however during the torsion and looping process, when the SHF derived structures are been recruited proliferation is more important. Our findings along with those of troponin I and Sox-9 immunodetection in the explants of the straight heart tube (Fig. 3) not only support our idea that at least a part of the AV canal was developed *in situ* by proliferation at the caudal border of PS of the straight heart tube, but also indicate that the initial development of FHF derived SHT (HH10), occurs mainly due to hypertrophy of cardiomyocytes, as previously mentioned [46,47]. In contras when the SHF derived structures are formed, the importance of proliferation increases, leading to the subsequent expansion of the ventricular cavities via a mechanism similar to the ballooning model proposed by Christoffels et al [17].

**New model of segmental patterning of the heart tube**

Based on our *in vivo* labeling findings, we propose a new model of segmental patterning of the heart tube during torsion and looping that differs from the currently accepted model (Fig 5). Initially, the FHF is represented by the straight heart tube, whereas the pharyngeal splanchnic mesoderm below the cephalic, caudal, and lateral limits of the straight heart tube corresponds to SHF [1,8-11]. The heart tube at the HH10 stage is formed by two segments (Fig 5A). AS is involved in IVS development (Fig 5A, F) but not in RV formation, as previously proposed [4]. The bifurcated PS is involved in the development of LV and proximal part of the AV canal (Fig 5A,F) but not in the atria formation [6]. New segments emerge as the SHF splanchnic mesoderm is recruited to the cardiac tube. At the cephalic border of the C loop is distinguished the beginning of the development of the segment classically called the conus (Fig 5B), which actually corresponds to the RV primordium [26,37]. Meanwhile, at the caudal end of the C-loop, the FHF-derived AV canal precursor with a tubular shape and the SHF-derived right and left primitive atria with a still bifurcated appearance are now present (Fig 5B, F). In the S loop (HH14), the RV and LV primordia as well as the IVS precursors begin to descend, whereas the primitive atria are more noticeable (Fig 5C, F). Later, in the U loop (HH16), the distal segment of the conus is still being recruited [26]. The incipient RV primordium and the well-developed LV primordium, which are separated by the IVS precursor, occupy a caudal position, whereas the primitive atria exhibit a dorsocephalic arrangement (Fig 5D, F). In the middle stage of cardiac septation (HH24), it has been reported the myocardial walled truncus is also manifested which, according to Sanchez-Gomez et al. (2005) [48], form the aortic and pulmonary valves with its insertion ring (Fig 5E, F). At this stage, the conus exhibits greater development, whose prospective fate, as recently proposed by Lazzarini et al. (2018) [37] is RV and the pulmonary infundibulum (Fig. 5E, F). Based on the findings of this study and the fact that the aortic sac shows vascular constitution from the beginning of development and is separated by neural crest cells in the pulmonary and aortic conduits, we speculate that the aortic sac is the aortic and pulmonary trunk precursor (Fig 5F).

**Fig 5. New Proposal for the Segmental Pattern of the Heart Tube during Torsion and Looping.** (A) Straight heart tube. (B) “C” loop. (C) “S” loop. (D) “U” loop. (E) Middle stage of cardiac septation. (F) Mature heart. Anterior segment of the straight heart tube corresponds to the primordium of the interventricular septum (IVSp). Posterior segment is composed of precursors of the left ventricle (LV) and atrioventricular canal (AVC). The segment classically considered the conus of the C-looped heart, actually participates in the complete right ventricle (RV) development. The region previously considered the RV primordium actually participates in the development of the middle and apical thirds of the interventricular septum. The supposed left ventricular primordium actually contributes cell populations to the development of LV and AV canal. Abbreviations: AVCp, atrioventricular canal primordium; C, conus; LA, left atrium; LVp, left ventricle primordium; RA, right atrium; RVOF, right ventricle outflow; PLA, primitive left atrium; PRA, primitive right atrium; RVp, right ventricle primordium; Sig, sigmoid valves; T, truncus.

**Conclusions**
Our findings regarding the prospective fate of FHF-derived straight heart tube and novel developmental patterning of FHF and SHF provide a frame of reference for future genetic and molecular studies aiming at uncovering the causes of congenital heart disease that involve the ventricles and IVS. From the anatomo-functional point of view, the differential origin of LV (FHF) with respect to RV (SHF) is important information to consider when developing surgical strategies for the correction of pathologies that involve the ventricular cavities.

**Acknowledgments**

The authors want to thank Mr. Oscar Ariel Alexis Ramirez, Mario Jauregui Castro, and Jorge Arturo Hilario for technical assistance.

**References**

1. Buckingham ME, Meilhac S, Zaffran S. Building the mammalian heart from two sources of myocardial cells. Nat Rev Genet. 2005;6(11):826-835.
2. Cui C, Cheuvront TJ, Lansford RD, Moreno-Rodriguez RA, Schultheiss TM, Rongish BJ. Dynamic positional fate map of the primary heart-forming region. Dev Biol. 2009;332(2): 212-222.
3. Ivanovitch K, Temiño S, Torres M. Live imaging of heart tube development in mouse reveals alternating phases of cardiac differentiation and morphogenesis. Elife. 2017;6: e30668.
4. De la Cruz MV, Sánchez-Gómez C, Palomino MA. The primitive cardiac regions in the straight tube heart (Stage 9-) and their anatomical expression in the mature heart: an experimental study in the chick embryo. J Anat. 1989;165:121-131.
5. Moreno-Rodríguez RA, Krug EL, Reyes L, Villavicencio L, Mjaatvedt CH, Markwald RR. Bidirectional fusion of the heart-forming fields in the developing chick embryo. Dev Dyn. 2006; 235 (1): 191–202.
6. Abu-Issa R and Margaret L. Kirby. Patterning of the heart field in the chick. Dev Biol. 2008;319(2): 223-233.
7. Colas JF, Lawson A, Schoenwolf GC. Evidence that translation of smooth muscle alpha-actin mRNA is delayed in the chick promyocardium until fusion of the bilateral heart-forming regions. Develop Dyn. 2000;218(2):316-330.
8. Mjaatvedt CH, Nakaoka T, Moreno-Rodriguez R, Norris RA, Kern MJ, Eisenberg CA, et al. The outflow tract of the heart is recruited from a novel heart-forming field. Dev Biol. 2001;238(1): 97-109.
9. Waldo KL, Kumiski DH, Wallis KT, Stadt HA, Hutson MR, Platt DH, Kirby ML. Conotruncal myocardium arises from a secondary heart field. Development. 2001;128(16): 3179-3188.
10. Abu-Issa R, Waldo K, Kirby ML. Heart fields: one, two or more? Dev Biol. 2004; 272(2):281-285.
11. Kidokoro H, Yonei-Tamura S, Tamura K, Schoenwolf GC, Saijoh Y. The heart tube forms and elongates through dynamic cell rearrangement coordinated with foregut extension. Development. 2018;145(7).
12. Rochais F, Mesbah Karim, Kelly RG. Signaling pathways controlling second heart field development. Circ Res. 2009;104: 933-942.
13. Cai CL, Liang X, Shi Y, Chu PH, Pfaff SL, Chen J, Evans S. Isl1 identifies a cardiac progenitor population that proliferates prior to differentiation and contributes a majority of cells to the heart. Dev Cell. 2003;5: 877-889.
14. Brade T, Pane L, Moretti A, Chien K, Karl-Ludwig Laugwitz KL. Embryonic Heart Progenitors and Cardiogenesis. Cold Spring Harb Perspect Med. 2013;3:a013847.
15. Davis CL. Development of the human heart from its first appearance to the stage found in embryos of twenty paired somites. Contrib Embryol. 1927;19: 245-284.
16. De la Cruz MV, Muñoz-Armas S; Muñoz Castellanos L. Development of the chick heart. Johns Hopkons University, Baltimore, MD. 1972.
17. Christoffels VM, Habets PE, Franco D, Campione M, de Jong F, Lamers WH, et al. Chamber formation and morphogenesis in the developing mammalian heart. Dev Biol. 2000;223:266-278.
18. Männer J. The anatomy of cardiac looping: a step towards the understanding of the morphogenesis of several forms of congenital cardiac malformations. Clin Anat. 2009;22(1):21-35.
19. Zaffran S, Kelly RG, Meilhac SM, Buckingham ME, Brown NA. Right ventricular myocardium derives from the anterior heart field. Circ Res. 2004;95(3):261-268.
20. Abu-Issa R. Heart fields: Spatial polarity and temporal dynamics. Anat Rec. 2014;297(2):175-182.
21. Srivastava D. Making or breaking the heart: From lineage determination to morphogenesis. Cell. 2006;126(6):1037-1048.
22. Moorman A, Webb S, Brown NA, Lamers W, Anderson RH. Development of the heart: (1) formation of the cardiac chambers and arterial trunks. Heart. 2003;89:806-814.
23. Meilhac SM, Esner M, Kerszberg M. Oriented clonal cell growth in the developing mouse myocardium underlies cardiac morphogenesis. J Cell Biol. 2004; 164(1).
24. Kelly RG, Buckingham ME. The anterior heart-forming field: voyage to the arterial pole of the heart. Trends Genet. 2002;18(4):210-216.
25. Verzi MP, McCulley DJ, De Val S, Dodou E, Black BL. The right ventricle, outflow tract, and ventricular septum comprise a restricted expression domain within the secondary/anterior heart field. Dev Biol. 2005; 287(1):134-145.
26. Rana MS, Horsten NC, Tesink-Taekema S, Lamers WH, Moorman AF, Van den Hoff MJ. Trabeculated right ventricular free wall in the chicken heart forms by ventricularization of the myocardium initially forming the outflow tract. Cir Res. 2007;100: 1000-1007.
27. Evans SM, Yelon D, Conlon DF, Kirby ML. Myocardial lineage development. Circ Res. 2010;107(12): 1428-1444.
28. Laugwitz KL, Moretti A, Lam J, Gruber P, Chen Y, Woodard S, et al. Postnatal isl1+ cardioblasts enter fully differentiated cardiomyocyte lineages. Nature. 2005;433(7026): 647-653.
29. Galli D, Domínguez JN, Buckingham M. Atrial myocardium derives from the posterior region of the second heart field, which acquires left-right identity as Pitx2c is expressed. Development. 2008;135: 1157-1167.
30. Devine WP, Wythe JD, George M, Koshiba-Takeuchi K, Bruneau BG. Early patterning and specification of cardiac progenitors in gastrulating mesoderm Elife. 2014;3.
31. De la Cruz MV, Castillo MM, Villavicencio LA, Valencia A, Moreno-Rodriguez R. Primitive interventricular septum, its primordium, and its contribution in the definitive interventricular septum: in vivo labeling study in the chick embryo heart. Anat Rec. 1997;247: 512-520.
32. De la Cruz MV, Moreno-Rodriguez R. Embryological development of the apical trabeculated region of both ventricles. The contribution of the primitive interventricular septum in the ventricular septation. In: De la Cruz MV and Marwald RR, editors. Living morphogenesis of the heart. Boston: Birkhauser;1998. Pp.121-130.
33. Bruneau BG. Signaling and transcriptional networks in heart development and regeneration. Cold Spring Harb Perspect Biol. 2013;5(3): a008292.
34. Hamburger B, Hamilton HL. A series of normal stages in the development of the chick embryo. J Morphol. 1951;88: 49-92.
35. Chapman SC, CollignonJ, Schoenwolf GC, Lumsden A. Improved method for chick whole-embryo culture using a filter paper carrier. Dev Dyn. 2001;220(3):284-289.
36. De la Cruz MV, Sanchez Gomez C, Arteaga MM, Argüello C. Experimental study of the development of the truncus and the conus in the chick embryo. J Anat. 1977;123: 661-686.
37. Lazzarini R, Gomez-Quiroz LE, Gonzalez-Marquez H, Villavicencio-Guzman L, Salazar-Garcia M, Sanchez-Gomez C. The proximal segment of the embryonic outflow (conus) does not participate in aortic vestibule development. Plos One. 2018;13(12): e0209930.
38. Lazik A, Liu Y, Sangiorgicand F, Maxon R. A method for preparing DiI-labeled tissue that allows single-cell resolution with conventional epifluorescence microscopy. Technical Tips Online. 1997;2:118-120.
39. Streeter GL. Development horizon in human embryos. Description of age group XV, XVI, XVII, and XVIII, being the third issue of the Carnegie Collection. Contrib. Embryol. 1948;32: 133-203.
40. Goor DA, Edwards JE, Lillehei CW. The development of the interventricular septum of the human heart; correlative morphogenetic study. Chest. 1970;58(5):453-467.
41. Harh JY, Paul MH. Experimental cardiac morphogenesis. I. Development of the ventricular septum in the chick. J Embryol Exp Morphol 1975;33(1): 13-28.
42. Ben-Shachar G, Arcilla R, Lucas R, Manasek F. Ventricular trabeculations in the chick embryo heart and their contribution to ventricular and muscular septal development. Circ Res. 1985:57: 759-766.
43. Franco D, Christoffels VM, Meilhac SM, Vincent M, Kispert A, Buckingham M, et al. Left and right ventricular contributions to the formation of the interventricular septum in the mouse heart. Dev Biol. 2006;294(2): 366-375
44. Contreras-Ramos A, Sanchez-Gomez C, Garcia-Romero HL, Cimarosti LO. Normal development of the muscular region of the interventricular septum. I. The significance of the ventricular trabeculations. Anat Histol Embryol. 2008;37(5):344-351.
45. van den Berg G, Moorman AFM. Concepts of cardiac development in retrospect. Pediatr Cardiol.2009;30:580-587.
46. Soufan A T, van den Berg G, Ruijter JM, de Boer PAJ, van den Hoff M J B, Moorman AFM, Regionalized Sequence of myocardial.cell growth and proliferation characterizes early chamber formation. Cir Res. 2006; 99(5):545–552.
47. Yunfei Shi, Jiang Yao, Gang Xu, Larry A Taber. Bending of the looping heart: differential growth. J Biomech Eng. 2014 Aug; 136(8): 0810021–08100215.
48. Sanchez Gomez C, Pliego PL, Contreras A, Munguia Rosas MA, Salazar GM, Garcia Romero HL, et al, Histological study of the proximal end distal segments of the embryonic outflow tract and great arteries. Anat Rec A Discov Mol Cell Evol Biol. 2005;283(1):202-211.
